# Strengthening the global network for sharing of marine biological collections: recommendations for a new agreement for biodiversity beyond national jurisdiction

**DOI:** 10.1093/icesjms/fsaa227

**Published:** 2020-12-13

**Authors:** Jane Eva Collins, Muriel Rabone, Thomas Vanagt, Diva J Amon, Judith Gobin, Isabelle Huys

**Affiliations:** 1 ABSint, Bruges, Belgium; 2 Faculty of Pharmaceutical Sciences, Clinical Pharmacology and Pharmacotherapy, KU Leuven, Leuven, Belgium; 3 Life Sciences Department, Natural History Museum, London, UK; 4 Department of Life Sciences, University of the West Indies, St. Augustine, Trinidad and Tobago

**Keywords:** areas beyond national jurisdiction, benefit sharing, biodiversity, biorepositories, capacity building, collections, deep sea, knowledge transfer, marine genetic resources

## Abstract

Biological collections are fundamental to marine scientific research and understanding of biodiversity at various scales. Despite their key importance, sample collections and the institutes that house them are often underfunded and receive comparatively little attention in the discussions associated with global biodiversity agreements. Furthermore, access to collections can be limited by inadequate systems, infrastructure, and networks. With negotiations underway for a new implementing agreement on biodiversity beyond national jurisdiction, marine genetic resources (MGR), including questions on the sharing of benefits, remains the most debated and contentious element. Disparities remain between States regarding access to and utilization of marine biological samples (including MGR) from areas beyond national jurisdiction. Addressing capacity gaps related to collections could provide a point of agreement during negotiations and enhance global inclusivity in access to and utilization of MGR. Here, we examine both existing capacity and regional gaps in marine collections. We propose the strengthening of a distributed network of marine biological collections, building on existing initiatives and emphasizing best practices to bridge regional gaps. Our recommendations include: promoting scientific best practice for the curation of collections; alignment with ocean observing, and sampling initiatives; a potential pairing scheme for collections in developing and developed States; raising awareness of collections and benefits to marine science including through a global registry/directory; and promoting sustainable funding mechanisms to support collections and sustain global generation of contributors and users.

## Introduction

The marine environment is vast and covers ∼70% of the Earth’s surface (Fenical and Jensen, 2006), with 95% of the total ocean volume considered areas beyond national jurisdiction (ABNJ) (see the Global Environmental Facility: https://www.thegef.org/topics/areas-beyond-national-jurisdiction—accessed 25 February 2020). Environments in ABNJ, largely comprised of deep ocean, remain poorly known, with up to 90% of species still undescribed (Ramirez-Llodra, 2010; Appeltans *et al.*, 2012; Poore *et al.*, 2015). Access to marine scientific samples is key to our collective understanding of biological diversity, an urgent need in light of increasing environmental change and the need for more effective conservation measures. However, not all countries currently have the required capacity to access collections (Juniper, 2013). Scientists cannot conduct biodiversity research unless they have access to the resources they need to investigate, as well as the ability to share those resources and their expertise (Prathapan *et al.*, 2018). The ability of States to develop and maintain in-country marine biological collections is therefore critical.

There is an opportunity to respond to this challenge in the negotiations underway at the United Nations: to develop an international legally binding instrument for the conservation and sustainable use of marine biological diversity of areas beyond national jurisdiction (BBNJ) under the auspices of the Law of the Sea Convention (UNCLOS, 1982). Negotiations address a “package” of elements [negotiations address a “package” of elements, including cross-cutting issues, as agreed during preparatory committee meetings in 2011 (UNGA Res. 69/292, UN Doc. A/Res/69.292, 6 July 2015, para. 2)]: marine genetic resources (MGR), including questions on the sharing of benefits; measures such as area-based management tools; environmental impact assessments; and capacity building and the transfer of marine technology (CB/TT). In order for the new agreement to be fair and effective, all States and stakeholders should have the opportunity to take part in the long-term sustainable use, management, and protection of the marine environment in ABNJ (Mohammed, 2017; BBNJ, 2019; Blasiak *et al.*, 2020). MGR and sharing of potential benefits derived thereof have proven to be the most contentious element of the treaty negotiations, with few detailed solutions proposed to date (Harden-Davies, 2017; Broggiato *et al.*, 2018; Voigt-Hanssen, 2018; Humphries *et al.*, 2020).

Long-term archiving of samples (whether biological, geological, or environmental) is key to the reproducibility of research. For example, archiving of voucher specimens post-analysis provides vital reference material to validate taxonomy (Hubert and Hanner, 2015; Funk *et al.*, 2017). Archiving also futureproofs collections for potential applications that are currently unknown, and maximizes the value of *in situ* sampling, particularly important for deep-sea cruises that are undertaken at vast expense (Glover *et al.*, 2018). With rapidly developing capabilities in genomics, such as environmental DNA monitoring and *in situ* sequencing, archiving is essential to provide reference libraries to understand findings (Glover *et al.*, 2018; Levin *et al.*, 2019). However, despite the fundamental importance of collections and biorepositories for research, they have received comparatively little attention in BBNJ negotiations and the literature, especially compared to ocean data, ocean observing, and related data practices (e.g. Levin *et al.*, 2019; Pearlman *et al.*, 2019; Snowden *et al.*, 2019; Thaler and Amon, 2019). Information regarding existing collections capacities, from areas within and beyond national jurisdiction, could therefore inform questions pertaining to MGR in these negotiations.

Biological collections or biorepositories can be broadly grouped as either clinical or non-clinical. The former as focused on human-health applications are not addressed in this article. The majority of non-clinical biorepositories are publicly funded, openly accessible biological collections, housed in large national museums and small university/government research institute museums, and working reference collections (Droege *et al.*, 2014). Natural-history collections are often highly heterogeneous, e.g. housing both marine and terrestrial collections with highly varied storage and preservation methods (Ali and Trivedi, 2011; Droege *et al.*, 2014). Collections may have defined applications (sometimes housed within a larger institute) such as longitudinal environmental monitoring, cryopreservation for conservation biology (Hagedorn *et al.*, 2017), or aquaculture (Martínez-Páramo *et al.*, 2017). Private-sector environmental or biodiversity biobanks often focus on biotechnology applications (Burgin, 2008; Rosendal *et al.*, 2016). In the public sector however, many collections face the existential threat of inadequate funding, threatening survival (Dupérré, 2020). Collections in developing countries are at even greater risk. Furthermore, in terms of discoverability of collections, while directories for natural-history collections exist, e.g. GRSciColl (Schindel *et al.*, 2016), they are in need of significant development (Hobern *et al.*, 2020), hampering discoverability.

The disparity between States to access and use marine biological samples (including MGR) from ABNJ stems from a variety of factors, including differences in access to and knowledge of collections, capacity to conduct marine scientific research (MSR), and access to funding. Here, we identify regional gaps and discuss how these may be addressed through benefit sharing and CB/TT in the BBNJ negotiation process (Rosendal *et al.*, 2016). We discuss best practices for the management of marine biological collections and provide perspectives on strengthening a network of collections by building cooperation between existing initiatives. Through the BBNJ agreement, promoting access to marine biological collections will enhance local, regional, and global understanding of marine biodiversity, its conservation, and its sustainable use.

## Methods

Information on institutions publishing datasets on the Ocean Biogeographic Information System (OBIS) (OBIS, https://obis.org—accessed 31 March 2020) was extracted using the OBIS application programming interface (API) (OBIS API, https://api.obis.org—accessed 31 March 2020) and analysed using R, version 3.5.3 (2019-03-11, R Core Team, 2019) in March 2020 (Supplementary File S1). In addition, institutes housing deep-sea collections from ABNJ were collated from records published to OBIS (https://mapper.obis.org/?areaid(=1&startdepth(=500)—accessed 20 September 2020) using the DarwinCore term “institutionCode” to collate the list of institutes. Here, records based on specimens only were included (no observational records or occurrences without voucher specimens) and were checked to ensure the records referenced a specimen voucher. Additional biorepositories and marine research institutes (including those which host live cell/culture collections) that have not published data to OBIS were found via Internet searches using recognized sources including the Global Genome Biodiversity Network (GGBN) (GGBN, http://www.ggbn.org/ggbn_portal/—accessed 30 March 2020) and the European Marine Biological Resource Centre (EMBRC) (EMBRC, http://www.embrc.eu—accessed 30 March 2020) between January and March 2020. Search keywords included: marine, biological, sample, material, collection, biobank, biorepository, and network. All institutions were georeferenced using the Ocean-Expert API (OceanExpert-API, https://github.com/iodepo/OceanExpert-API—accessed 30 March 2020) (or Google Maps if not recorded in OBIS) and plotted using QGIS, Version 3.10, Coruña (QGIS.org, 2020). A scoping literature review was also undertaken in January and February 2020 to gather information regarding CB/TT requirements for marine biological collections and biorepositories. Search keywords included: capacity building, marine, biological, sample, material, collection, biorepository, and network. Data sources included PubMed, Embase, Google Scholar, and EurLex.

## Regional gaps in marine biological collections

**Figure 1.**
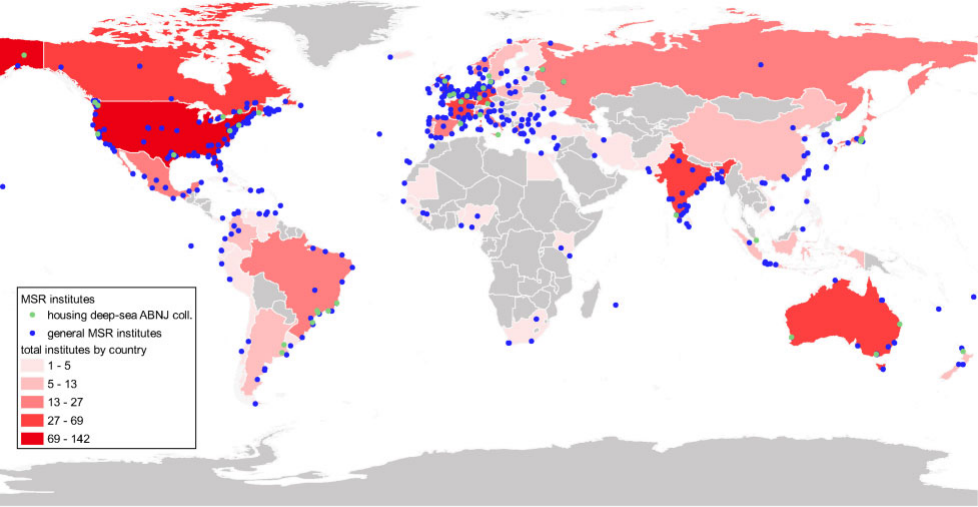
Location of MSR institutes holding collections/marine collections, including those specifically housing deep-sea collections from ABNJ (where associated data are published to OBIS), as listed in Supplementary Table S1.

In total, 785 institutes in 76 countries were recorded, including both primarily collection-focused MSR institutes (e.g. museums or biorepositories) and those with collection holdings within a wider institute, e.g. academic or government-run institutes/facilities/organizations holding reference collections (Figure 1 and Supplementary Table S1). Here, the term “collections” is used to encompass both collection-focused MSR institutes such as formal biobanks/biorepositories and museums, and the collection holdings of (non-collection-focused) MSR institutes. The majority was publicly funded, but privately funded collection organizations were also evident (Supplementary Table S1). These collections are primarily evident in countries of the Global North including the United States and Europe and globally are concentrated in coastal regions, particularly outside Europe. The highest numbers were found in the United States by a significant margin (142 in total, with the next highest number in Canada at 69, Figure 1). There is a particular lack of marine collections in countries along the northern, eastern, and southwestern coasts of Africa, with none evident in Cameroon or Angola, for example and also the Arabian Peninsula, much of Southeast Asia, and Pacific Island countries (Hawaii and New Calendonia being exceptions). In addition, there are very few along the southwestern and northern coasts of South America, in Central-American countries south of Belize, and smaller Caribbean States (Figure 1).

These gaps are consistent with long-standing disparities in scientific capacity between the Global South and North (Ladle *et al.*, 2015; Velasco *et al.*, 2015). However, these gaps potentially extend beyond the absence of biorepositories. Furthermore, evidence of MSR institutes with collections may not necessarily equate to capacity to utilize those collections. This assessment focused on collections with a digital presence, likely resulting in an underestimation and biasing of results towards those in the Global North. It is important to emphasize that this assessment is non-comprehensive, for example institutes publishing collections records to GBIF (Global Biodiversity Information Facility) or the collections registry GRSciColl (Schindel *et al.*, 2016) were not included given complexities of separating marine and terrestrial collections from these vast global datasets. Furthermore, mapping MSR institutes does not equate to marine collections/available collections—while the vast majority of MSR institutes house collections, a spectrum exists from collections focused institutes such as museums and biobanks, or university museums (part of larger MSR organizations) to institutes with small working collections without long-term archiving processes/procedures and collections with very limited wider availability or usage.

Inclusion in the present study of a subset of MSR organizations that house collections from ABNJ is also non-comprehensive, including only those organizations that publish data to OBIS. In-depth analysis of global collections capacity is hampered somewhat by the lack of visible collection-level data as opposed to specimen-level data that are published on OBIS (and GBIF). However, here, we provide an overall picture of locations of MSR institutes with marine biological collections that can be developed as work at the Taxonomic Databases Working Group (TDWG) and GBIF, for example on developing collections descriptions progresses (Thessen *et al.*, 2019).

## Challenges facing existing collections

Many collections, particularly publicly funded biorepositories, museums, and collections housed in academic research institutes face a number of challenges. As many collections are maintained long term and often in perpetuity, particularly in museums, and include an array of associated costs such as equipment, consumables, infrastructure, maintenance and staff time, biorepositories require sustainable, long-term funding (Rosendal *et al.*, 2016). However, they are regularly underfunded, particularly in countries with fewer available resources and infrastructure. This issue is further amplified for deep-sea research, where the immense fieldwork costs result in proportional reductions for post-cruise work, including archiving (Glover *et al.*, 2018). These funding issues partly stem from under-valuing of curatorial work in science, with greater recognition for activities such as grant applications and publications (Thessen *et al.*, 2019). Curation is closely linked to taxonomy, which has seen an existential crisis in recent decades (Dupérré, 2020).

A compounding issue is that museums and biorepositories are at the coalface of new and emerging legal requirements associated with international biodiversity agreements. For areas within national jurisdiction, where provisions under the Nagoya Protocol apply, it is becoming increasingly apparent that compliance with current access and benefit sharing (ABS) frameworks can be challenging and burdensome within some national jurisdictions (Nagoya Protocol, 2011; Prathapan *et al.*, 2018; Humphries *et al.*, 2020; Laird *et al.*, 2020). During BBNJ negotiations, approaches under consideration to govern MGR and benefit sharing have to date involved elements of existing ABS frameworks, which may be unsuitable for the vast interconnected ABNJ system, lacking a “provider” State (Greiber, 2012; Oberthür and Pożarowska, 2013; Laird *et al*., 2020). The call for a revised approach, including an emphasis on global cooperation and direct involvement of the scientific community in the BBNJ negotiation process, should be heeded (Humphries *et al.*, 2020; Laird *et al.*, 2020).

Several articles in the draft BBNJ treaty text have direct implications for biorepositories. Article 10 (2019) [article 10—[Collections of] [and] [Access to] marine genetic resources of areas beyond national jurisdiction (BBNJ revised draft text November 2019)] requires that States ensure that samples, data, and information related to MGR collected in ABNJ are deposited in open-source databases, repositories, or gene banks, within a limited timeframe. Article 11.3(b) [article 11—[Fair and equitable] sharing of benefits (BBNJ revised draft text, 2019)] describes making MGR samples, data, and related information openly accessible as a form of non-monetary benefit sharing. Archiving and open access to collections is of course critical; however, an unfunded obligation for public institutions to receive, curate, and provide access to collections is not sustainable. Museums and biorepositories are working beyond capacity and already subsidize non-monetary benefits in this way (Rabone *et al.*, 2019). Unrestricted access to samples (unlike data) is also complicated by their finite nature, given that they erode with usage. Therefore, policies to maximize the sustainability of usage are critical. Proposed draft articles to make MGR-related information available within a given timeframe are also complicated by the fact that many collections will not be fully sorted or identified for extended periods, taxonomy being a very time-intensive process.

## Supporting best practices for marine biological collections

Evidence-based best practices result in high-quality specimens for future research, maximizing the value and potential applications for collections. International agreements could be leveraged to increase adoption of best practice, coordination, and knowledge sharing (Campbell *et al.*, 2018). Here, we outline how existing best practices could be better distributed through existing networks and initiatives.

### Sample collection, archiving, and sharing

Best practices for sample collection and storage will depend on the type/taxa of material in question and the intended use, e.g. RNA extraction requires storage of samples at −80°C (McCloud, 2010; Benson *et al.*, 2013; Hagedorn *et al.*, 2017; Xiao *et al.*, 2018) [Procedure for Curation of Routine Microbiological Sample, adopted by the Integrated Ocean Drilling Program (IODP), Center for Deep Earth Exploration (CDEX) and Kochi Core Center (KCC)—http://www.kochi-core.jp/iodp-curation/publication_file/RMS%20Procedure%20ver.1.9.pdf—accessed 04 March 2020]. There is extensive literature available on best practices in collection management: from sampling, collection archiving, and sharing, to data management and community involvement (McCloud, 2010; Benson *et al.*, 2013; Glover *et al.*, 2015; Kopf *et al.*, 2015; ten Hoopen *et al.*, 2015; Vaught, 2015; Clark *et al.*, 2016; Martínez-Páramo *et al.*, 2017). Many best-practice guidelines exist therefore, but they are distributed across different sources. This information should be Findable, Accessible, Interoperable and Reusable (FAIR; Wilkinson *et al.*, 2016), and the Ocean Best Practices System (OBPS) [OceanBestPractices maintained by the International Oceanographic Data and Information Exchange (IODE) of the Intergovernmental Oceanographic Commission of UNESCO, https://www.oceanbestpractices.net—accessed 01 April 2020] has addressed this through development of a platform for sharing and accessing MSR best-practice documents (Pearlman *et al.*, 2019). This system ensures that the contents of best-practice documents are discoverable and that new methodologies and protocols are dynamically updated (OceanBestPractices maintained by the International Oceanographic Data and Information Exchange (IODE) of the Intergovernmental Oceanographic Commission of UNESCO, https://www.oceanbestpractices.net—accessed 01 April 2020). There is significant scope for the biobanking and museum sectors to adopt and utilize the OBPS platform.

The development of best-practice guidelines for the research community, which enhance the exchange of data/material, also promote collaboration amongst biorepositories [Global Genome Biodiversity Network (GGBN) Guidance, 2015; Droege *et al.*, 2016; Nussbeck *et al.*, 2016]. To join GGBN, for example specific sample and data curation standards must be met [Global Genome Biodiversity Network (GGBN) Guidance, 2015]. The collections are then discoverable through the GGBN Data Portal, supporting access to samples held within the network (Droege *et al.*, 2016) (https://wiki.ggbn.org/ggbn/About_GGBN#Making_Genomic_Collections_Discoverable_for_Research_through_a_Networked_Community_of_Biodiversity_Repositories—accessed 13 April 2020). To standardize the methods used for curation of collections, particularly when samples fall under the Nagoya Protocol (2011), the Consortium of European Taxonomic Facilities (CETAF) has established an Access and Benefit-Sharing Code of Conduct and Best Practices (https://cetaf.org/sites/default/files/documents/cetaf_abs_code_of_conduct_all_annexes.pdf—accessed 13 April 2020), providing a set of basic collection-management principles. A similar framework may prove useful within the BBNJ context.

In terms of collections loans and sharing, marine collections may hold samples originating from both within and beyond national jurisdiction, resulting in varying procedures and requirements for access. Samples collected within national jurisdictions will require Material Transfer Agreements (MTAs) when ownership is transferred between institutes. Best-practice procedures involving MTAs have been developed under (draft) national ABS regulations and in compliance with the Nagoya Protocol and implemented at institutions, e.g. at Marbank (Rosendal *et al.*, 2016). Procedures that will apply when users request access to *ex situ* ABNJ samples in collections are yet to be determined and could be informed by the outcome of BBNJ negotiations, and potentially also by discussions under the CBD Conference of the Parties (2019–2020 intersessional period, DSI on genetic resources, https://www.cbd.int/dsi-gr/2019-2020/—accessed 01 April 2020). It is critical that legislation does not hamper the ability to do scientific work and share collections and data (Rabone *et al.*, 2019). Funding to enable sharing should be considered; some European museums, for example have a policy of cost recovery for loans, with waivers for researchers from developing countries.

Long-term funding streams are also needed to develop and facilitate best practices. Examples include the EU Green Fund (The EU Green Fund, https://ec.europa.eu/info/business-economy-euro/banking-and-finance/green-finance_en—accessed 29 March 2020), the EU Post-2020 Biodiversity Strategy (The EU Post-2020 Biodiversity Strategy, https://ec.europa.eu/environment/nature/biodiversity/strategy/index_en.htm—accessed 29 March 2020), the Crop Trust (Crop Trust—https://www.croptrust.org/—accessed 13 April 2020), and the associated Svalbaard Global Seed Vault (Norway). More focused and innovative solutions (such as public–private partnerships) are needed in developing countries where there are even larger fundamental gaps, such as basic infrastructure and trained museum personnel. In the field of tropical parasitology, European research councils and funding organizations are committing grant calls for in-country facilities and collaborations, for example (Wellcome Trust includes grant calls for in-country facilities).

### Networks and alignment of existing best practices

Networks are crucial for knowledge exchange, for building collaborations on local, regional, and global scales, and for the development and dissemination of best practice. Some existing biobanking networks provide significant opportunities for cooperative, multidisciplinary activities to develop networks of marine biological collections, including GGBN (Droege *et al.*, 2016), the International Society for Biological and Environmental Repositories (ISBER) (ISBER, https://www.isber.org—accessed 30 March 2020), and the European Middle Eastern & African Society for Biopreservation and Biobanking (ESBB) (ESBB, https://esbb.org—accessed 30 March 2020) (Table 1). These networks and initiatives can be further strengthened and mobilized via the BBNJ agreement. While including some important marine collections, e.g. Ocean Genome Legacy (https://wiki.ggbn.org/ggbn/Global_Genome_Biodiversity_Network-Global_Genome_Initiative_Awards_Program#Institution:_Ocean_Genome_Legacy_Center—accessed 30 March 2020), collections in GGBN are primarily terrestrial. There is potential, therefore, to promote linkages of GGBN with ocean-focused initiatives [Deep Ocean Observing Strategy (DOOS), for example].

**Table 1.** Examples of networks/initiatives that foster collaborative MSR, relevant to strengthening a global network of marine biological collections.

Networks/initiatives	Description
Biobank focused	
GGBN	An international network of institutions that share an interest in long-term preservation of genomic samples representing the diversity of non-human life on Earth
ISBER	A global forum that addresses harmonization of scientific, technical, legal, and ethical issues relevant to repositories of biological and environmental specimens
EMBRC	A global reference research infrastructure responding to the societal grand challenges through advanced marine biology and ecology research
World Federation for Culture Collections	The organization aims to promote and support the establishment of culture collections and related services and to set up an information network between collections and users
ESBB	Provides information on biobanking to members and beyond to advance biosharing and help solve global challenges
Museum focused	
CETAF	A taxonomic research network contributing to Europe’s knowledge-base by enhancing synergies across member collections and research capabilities
Society for the Preservation of Natural History Collections	An international society aiming to improve the preservation, conservation, and management of natural history collections
DiSSCo	A European research infrastructure for natural science collections, to support the digital unification of all European natural science assets under common curation and access policies
Ocean observing-focused	
GOOS	A sustained collaborative system of ocean observations, encompassing *in situ* networks, satellite systems, governments, UN agencies, and individual scientists
DOOS	An initiative under the auspices of GOOS with the aim of coordinating and expanding deep-ocean observing efforts
OBPS	A permanent document repository that aims to provide a discovery point for research groups to find community accepted existing ocean best practices, to support the end-to-end best practices value chain

### Networks and a distributed collection approach

Collections are often distributed between institutes post-cruise completion, with specialists, for example identifying the fauna collected. This process has many benefits—it can promote best practice through usage of common data standards and interoperable data systems to facilitate sharing of samples and data (Hobern *et al.*, 2019). Furthermore, distributed collections also distribute the risk of loss. The recently established Distributed System of Scientific Collections (DiSSCo) (DiSSCo—https://www.dissco.eu—accessed 01 April 2020) is a European Research Infrastructure that aims to unify natural science collections with common curation policies and best practices, to ensure that collections and data are FAIR. Global genomic sampling programmes are also underway through the Earth BioGenome Project (Lewin *et al.*, 2018). Museums will play a key role in the development of resulting molecular collections. These initiatives offer an opportunity to streamline sampling efforts and maximize existing collections. There are existing and proposed global registries of collections, e.g. GRSciColl and the One World Collection (Owens and Johnson, 2019). However, they require further development to be fit-for-purpose with efforts for this ongoing (TDWG Collections Description Working Group, https://www.tdwg.org/community/cd/—accessed 20 April 2020) (Thessen *et al.*, 2019). The analysis conducted in the present study found that multiple steps are currently required to collate a georeferenced list of marine collections (Supplementary File S1). A dynamically updated and citeable collections registry, as currently in development (Thessen *et al.*, 2019), could be published on the proposed clearing house mechanism, strengthening awareness of existing collections, supporting transparency and discoverability of marine biological collections, and supporting MSR.

### Alignment with ocean observing initiatives

While not collections focused, ocean observing/monitoring initiatives do collect samples, e.g. the Global Ocean Observing System (GOOS) (GOOS, https://www.goosocean.org/—accessed 28 March 2020), the DOOS (DOOS, https://deepoceanobserving.org/—accessed 28 March 2020), and the Marine Biodiversity Observation Network (Marine BON) (Marine BON, https://marinebon.org—accessed 12 July 2020) (Bax *et al.*, 2018). By strengthening collaborations between ocean observing initiatives and the museums, collections resulting from ocean observing activities could be archived long-term for a range of additional research and applications, for example genomics. With sampling systems in place, these initiatives could be leveraged to broaden sampling in ABNJ, e.g. repurposing Argo floats to undertake longitudinal genetic monitoring. Likewise, utilizing ships of opportunity to undertake additional biological sampling could greatly reduce the expense of ship-time, particularly beneficial to developing countries where this presents a major barrier to deep-sea research (Levin *et al.*, 2019). Overlapping deep-sea sampling efforts should be avoided at all costs, considering the great investments involved.

### Facilitating CB/TT

Some networks of collections provide resources and tools for CB/TT. While clinical in focus, ISBER has a relevant “Best Practice” website, housing “recommendations for repositories” (Campbell *et al.*, 2018), and GGBN has a similar resource for all aspects of handling molecular collections. Other ISBER initiatives include the Self-Assessment Tool, to compare repository practices to ISBER best practices (Betsou, 2018). These resources help collections to strengthen their practices by identifying areas for improvement. Other capacity-building efforts include educational programmes to develop knowledge required for collections management. ESBB provides webinars on biobanking and biopreservation knowledge, highlighting innovations, alongside summer schools and accredited biobanking courses. Less developed countries require a more fundamental capacity-building effort, which may require regional efforts to achieve sustainability.

## Benefits of strengthening a global network of marine biological collections

All four elements of the BBNJ agreement rely on MSR to address knowledge gaps, which will be advanced through improved access to marine biological collections (Rabone *et al.*, 2019). Therefore, raising awareness of and building a network of marine collections can play a critical role in achieving a number of the BBNJ goals.

According to article 42 of the BBNJ revised draft text, “developing marine scientific and technological capacity” and “increasing, dissemination and sharing knowledge” are key objectives of CB/TT to support the conservation and sustainable use of BBNJ (BBNJ, 2019). Raising awareness and enhancing understanding of existing marine biological collections, observing and sampling efforts, and related best practices leveraging existing networks as above (e.g. GGBN, GOOS/DOOS) could support MSR. This could enable a greater number of scientists to access and study biological samples from the global marine environment, facilitating the generation and sharing of biodiversity knowledge (Harden-Davies, 2017; Broggiato *et al.*, 2018; Closek *et al.*, 2019; Collins *et al.*, 2019).

Strengthening a network of collections that hold biological samples from ABNJ could galvanize collaborative CB/TT efforts. This would require significant scientific knowledge, data and information, human and financial resources, specialist skills, and equipment (Harden-Davies, 2017; Rabone *et al.*, 2019; Harden-Davies and Snelgrove, 2020). A number of options regarding non-monetary and monetary benefit sharing have been proposed to date, but with few detailed solutions suggested (Voigt-Hanssen, 2018; BBNJ, 2019). Enhanced access to MGR from ABNJ via a network of distributed collections could facilitate collaborative MSR (Broggiato *et al.*, 2018; Collins *et al.*, 2019). A review of benefit-sharing options with the aim to build on and support a network of collections could prove useful for providing a point of consensus during negotiations, helping to progress towards an agreement of the package overall.

Article 44.4 (BBNJ, 2020) states that “capacity-building and the transfer of marine technology shall be based on and be responsive to the needs and priorities of developing States Parties”. As such, closing regional gaps through a network of collections (see Figure 1) would greatly increase accessibility and give States more ownership of their own material and research, enabling scientific research to meet local, regional, and global needs. Access to collections, capacity, and technology is critical for scientists to conduct research, especially a means for scientists in developing States to advance marine research and to improve the conservation of marine biodiversity within their own waters (and beyond) (Jaspars *et al.*, 2016; IOC, 2017; Closek *et al.*, 2019).

## Recommendations for BBNJ negotiations

By better understanding the existing capacity of marine biological collections (Figure 1) and the associated best practices, recommendations regarding CB/TT and benefit-sharing measures to be adopted under the BBNJ agreement can be made (see recommendations below). The concept of capacity building for the purpose of developing and connecting sample collections is supported by section 3 of UNCLOS (1982), and in particular articles 275 [Article 275. Establishment of national centres. (i) States, directly or through competent international organizations and the Authority, shall promote the establishment, particularly in developing coastal States, of national marine scientific and technological research centres and the strengthening of existing national centres, to stimulate and advance the conduct of marine scientific research by developing coastal States and to enhance their national capabilities to utilize and preserve their marine resources for their economic benefit. (ii) States, through competent international organizations and the Authority, shall give adequate support to facilitate the establishment and strengthening of such national centres so as to provide for advanced training facilities and necessary equipment, skills and know-how as well as technical experts to such States which may need and request such assistance.] and 276 [Article 276. Establishment of regional centre. (1) States, in coordination with the competent international organizations, the Authority and national marine scientific and technological research institutions, shall promote the establishment of regional marine scientific and technological research centres, particularly in developing States, to stimulate and advance the conduct of marine scientific research by developing States and foster the transfer of marine technology.], which reinforces the need to establish and strengthen both national and regional MSR centres. Different forms of CB/TT can be considered in the context of BBNJ negotiations, with a number listed in article 46 currently under consideration (UNLCOS, 1982; IOC, 2005; BBNJ, 2019; Harden-Davies and Snelgrove, 2020). Considering the divergence in perspectives regarding which measures to adopt and how these should be achieved, supporting a network of collections could help focus the specific forms of CB/TT and benefit sharing to meet collective objectives. Here, agreement could also assist in building overall consensus during BBNJ negotiations and subsequent implementation processes.

### Summary of recommendations to bridge regional gaps and support best practice in collections

- Develop a pairing scheme for biorepositories: the equivalent of mentorship pairing, but for developed and developing country biorepositories/museums, to facilitate training, mentorship, expert exchanges, collaborative scientific missions, and joint research projects/grant applications (Campbell *et al.*, 2018; Harden-Davies and Snelgrove, 2020). Our review of existing initiatives that foster collaborative research and best practices (as described in Table 1 and “Supporting best practices for marine biological collections” section) describe elements relevant to strengthening a global network of marine biological collections.
- Develop a network of distributed collections: the distribution of marine collections between institutions and countries, with data linked by shared or compatible databases/usage of global data standards such as Darwin Core (Hobern *et al.*, 2019; Thessen *et al.*, 2019). In-country repositories can be supported here by the pairing scheme and by distributing collections between institutes in developing and developed States, with appropriate resourcing to support establishment and maintenance of collections in-country (see “Supporting best practices for marine biological collections” section).
- Facilitate knowledge sharing: joint workshops, mentoring, educational courses, and training initiatives [aligning with existing initiatives such as Ocean Teacher (Ocean Teacher, https://classroom.oceanteacher.org—accessed 26 April 2020)] for collections/collections-based research could address knowledge required for all stages in the sample life-cycle: sampling, storage, curation, and down-stream analysis.
- Leverage existing networks and initiatives: networks and initiatives, e.g. GGBN, could facilitate cooperation between collections by supporting knowledge exchange and collaborations and by enhancing awareness and adoption of best-practice procedures. In addition, networks focused on ocean observing could be leveraged both to raise the awareness of sampling activities and opportunities for collaboration and capacity building (Levin *et al.*, 2019). Increased collaborations with ocean-observing networks and museums could lead to increased long-term archiving and secondary usage of samples collected through observing activities (see Table 1 and “Supporting best practices for marine biological collections” section above).
- Build awareness and adoption of existing MSR best practices: from sampling and sharing of marine biological samples, to data management and usage of global data standards, best practices should be developed to promote interoperability and sharing between institutions as well as sustainability of high-quality collections for future research (Rabone *et al.*, 2019). This could be facilitated by existing networks as described above (see Table 1) and platforms such as Ocean Best Practices.
- Develop a global registry of marine collections: to date, there is no single, comprehensive registry/directory of marine biological collections. A number of collections lack any form of associated online information, which renders samples archived in those institutions difficult to locate. As such, the development of a dynamically updated marine collections registry, aligned with existing efforts underway for global collections registries and made available on the proposed clearing house mechanism would support discoverability and sharing of samples between collections and therefore enhance opportunities for MSR (Schindel *et al.*, 2016; Thessen *et al.*, 2019). This would also enhance transparency and traceability for MGR.
- Facilitate sustainable funding: funders should recognize the critical importance of collections and long-term archiving to MSR and capacity building and support with sustainable long-term funding (Rosendal *et al.*, 2016). Long-term funding streams, similar to the EU Green Fund and the EU Post-2020 Biodiversity Strategy, could be adapted for this purpose. More focused and innovative solutions, such as grant calls for in-country collaboration, may also promote long-term archival and maintenance of collections.

## Conclusion

High-quality collections are critical to high-quality science, and accessible collections are fundamental to capacity building. While marine biological collections exist around the world, there are significant regional gaps. Concerted and coordinated efforts are needed to address these long-standing disparities and to strengthen a global network of collections. These efforts should be linked directly to existing global networks, such as GGBN and GOOS/DOOS, with the possibility to integrate existing initiatives and communities, such as global ocean observing networks. A focus during forthcoming BBNJ negotiations and subsequent implementation processes on supporting global networks of collections with a focus on less developed countries would support MSR and CB/TT efforts, with the additional benefit of providing a useful point of agreement to help progress multiple elements in the BBNJ package (and even other international agreements such as the Nagoya Protocol). Overall, this could lead to enhanced collective MSR efforts, in turn advancing scientific knowledge regarding marine biodiversity both beyond and also within national jurisdiction, thereby facilitating conservation and sustainable use as well as meeting other local, regional, and global needs.

## Supplementary data

Supplementary material is available at the *ICESJMS* online version of the manuscript.

## Data availability

The data underlying this article are available in the article and in its online supplementary material.

## Supplementary Material

fsaa227_Supplementary_Data
